# Methodological Aspects of the Potential Use of Dendrochronological Techniques When Analyzing the Long-Term Impact of Tourism on Protected Areas

**DOI:** 10.1371/journal.pone.0136830

**Published:** 2015-09-01

**Authors:** Szymon Ciapała, Paweł Adamski

**Affiliations:** 1 Department of Natural Environment Sciences, Faculty of Ecology, University School of Physical Education, Cracow, Poland; 2 Institute of Nature Conservation, Polish Academy of Sciences, Cracow, Poland; DOE Pacific Northwest National Laboratory, UNITED STATES

## Abstract

Intensification of pedestrian tourism causes damage to trees near tourist tracks, and likewise changes the soil structure. As a result, one may expect that annual amount of trees growing near tracks is significantly lower than deeper in the forest. However, during the study of the long-term impact of tourism on the environment (determined from tree increment dynamics), some methodological problems may occur. It is particularly important in protected areas where law and administrative regulations related to nature conservation force research to be conducted using small samples. In this paper we have analyzed the data collected in the Polish part of the Tatra National Park in the two study plots divided into two zones each: the area directly under the influence of the tourist's trampling and the control group. The aim of such analyses was to present the potential effects of the factors which may affect the results of dendrochronological analysis: (i) small size of samples that affects their representativeness, (ii) spatial differences in the rates of the process, as a result of spatial variability of environmental factors and (iii) temporal differences in the rates of the process. This study confirms that the factors mentioned above could significantly influence the results and should be taken into consideration during the analysis.

## Introduction

The impact of tourism on the natural environment has been comprehensively addressed in research for quite a long time [1,2]. This interest is caused by the ubiquity of tourism on the one hand [3] and the fact that environmentally attractive areas represent a group of very popular destinations on the other, which is partially caused by growing interest in ecotourism [4,5]. Out of all recreational areas, forests play a special role [6]. For example research conducted in Germany [7] has shown that 90% of German forest areas are fit for recreation without detriment to other functions of forests, namely: water protection, soil protection, and timber production. Furthermore, tourism is sometimes seen as a kind of safeguarding measure against more invasive forms of activity in environmentally valuable sites [8,9].

However, the question of determining the extent to which forest areas are affected by tourist use remains open, as different components of the natural environment show different sensitivity to tourist interference. If we limit our focus to the impact of pedestrian tourism, which is considered the least invasive form, we can see that vegetation damage conducive to soil erosion [10,2,11,12] and the impact on macrofauna [2] are the most thoroughly explored issues. Nonetheless, it should be stressed that the majority of available results pertain to short-term impacts, even in the case of erosive processes. Due to their variable intensity and the lack of systematic monitoring, there is often no data to determine when these processes started and how their intensity changed through time.

For this reason, the analysis of long-term tourist impact on the natural environment is in most cases based on combinations of data collected from different sites, which are subject to more or less intensive tourist pressure, or from the same areas at different time points [13,14,15]. Although such an approach appropriately explains the mechanism of tourist impact on the environment, it features one major restraint: while describing the consequences of the pressure associated with heavy tourist traffic, it does not allow one to determine whether this pressure changed with the intensity of tourist traffic in a given area and how. As trees grow at the same location for at least several dozen years, the analysis of their annual increments has long been used to monitor the changing conditions of the external environment [16,17,18,19,20,21,22,23]. Because soil compaction affects the properties of the sorption complex on tourist tracks and the intensification of tourist traffic results in the roots of trees adjacent to tracks being exposed and injured, the authors have assumed that these factors have a significant impact on the condition of trees growing at such sites. Therefore, it can be expected, that the relation between tourist traffic intensity and the widths of tree rings can be reconstructed on a similar basis.

Results of studies conducted in the Tatra National Park [24]show that the above-presented scheme actually functions, however, the use of traditional dendrochronological analyses involves a number of difficulties and methodological traps. For this reason, the aim of this paper is to present methodological problems identified by the authors while conducting field studies and processing data.

These problems refer to two major aspects:

### Sample size selection

The selection of sample size is one of the basic problems for all kinds of empirical research that involves statistical analysis of the results. According to the key principles of the probability calculus, while increasing the sample size, the parameters of its distribution gain an increasingly close resemblance to the parameters of the statistical population from which the sample has been drawn [25,26]. Based on duly adopted assumptions, it is therefore possible to both estimate the size of a representative sample and analyze the statistical power of tests applied [26,27,28]. Techniques for the estimation of required sample sizes are strictly related to the distribution parameters of analyzed variables, while the general rule indicates that the smaller the tested difference, the larger the sample size required [28,29,30].

In order to perform detailed estimations, it is necessary to adopt several assumptions concerning the: (1) satisfactory significance level, (2) types of variable distribution, (3) value of the expected difference, and (4) sample distribution parameters [31]. Whilst with regard to the significance level it is customary to adopt p = 0.05. Sometimes the information about the distribution parameters of the studied trait may be derived with high probability from the literature data. The predicted value of differences and the sample distribution parameters usually require a pilot study to be conducted. When doing research in protected areas, the extraction of increment cores, as an invasive method, requires permission from the nature conservation authorities. Such permission allows for a set specific number of samples, the amount of which needs to be established a priori. In such a situation, it is particularly important to ensure sample representativeness. Sometimes, however, due to law constrains on research taken in protected areas or on protected species, the sample size have to be minimalized. As is common in dendrochronological studies designed to reconstruct a specific environmental signal, we limited our sampling of trees to those without any obvious signs of damage or disease to maximize the likelihood of capturing the signal of interest in relation to other environmental signals also recorded in surrounding trees [18]. According to the hypothesis put forward by the authors, the use of a randomized method–i.e. drawing [32]–when having a pre-established sample size may have a negative impact on the representativeness of results obtained from small samples.

### Analysis of the process

Results of studies conducted to date [24] suggest a possibility that one of the negative consequences of tourist traffic on the environment is the reduced increment rate in trees growing in the direct vicinity of hiking trails in relation to trees growing beyond the reach of direct tourist impact. However, as tree ring sizes decrease with the advance of tree age, it is crucial for the testing of the hypothesis to ascertain whether the rate of this age-related reduction grows under tourist pressure. This may be determined through comparing regression lines for changes in tree ring sizes, especially their slope coefficients as their sign denotes the direction and their absolute value denotes the gradient of a regression line relative to the axis of abscissae. Parallel tests are used to determine whether processes described by two regression equations advance at the same rate [33]. However, the application of these tests may involve the occurrence of major artifacts resulting from the inhomogeneity of study groups. Samples collected in different fragments of the same area may differ in parameters not related directly with the impact of the studied factor. The effect of these parameters may prove stronger than the tourist impact. Moreover, a regression fit to the cloud of points in such situations may turn out too poor to derive reliable comparisons between regression lines. Another problem is caused by the fact that the character of analyzed changes is often curvilinear [34]. The aforementioned parallel tests are inapplicable in such cases. One way to solve this problem is to split the function’s domain into sections to which linear analysis may be applied [35]. However, there are no standardized criteria for this division, and the adoption of arbitrary values may significantly affect the results.

## Materials and Methods

We conducted our field-study in the Polish Tatra National Park. The climate in this region is changing simultaneously with increasing altitude above sea level. The altitude range, where the study was conducted, annual average air temperature ranges between 2 and 4°C, with rainfall around 1600 mm per year, resulting in 150 days of snow cover throughout the year. Permanent snow cover occurs at the beginning of November and disappears in late March.

**Fig 1:**
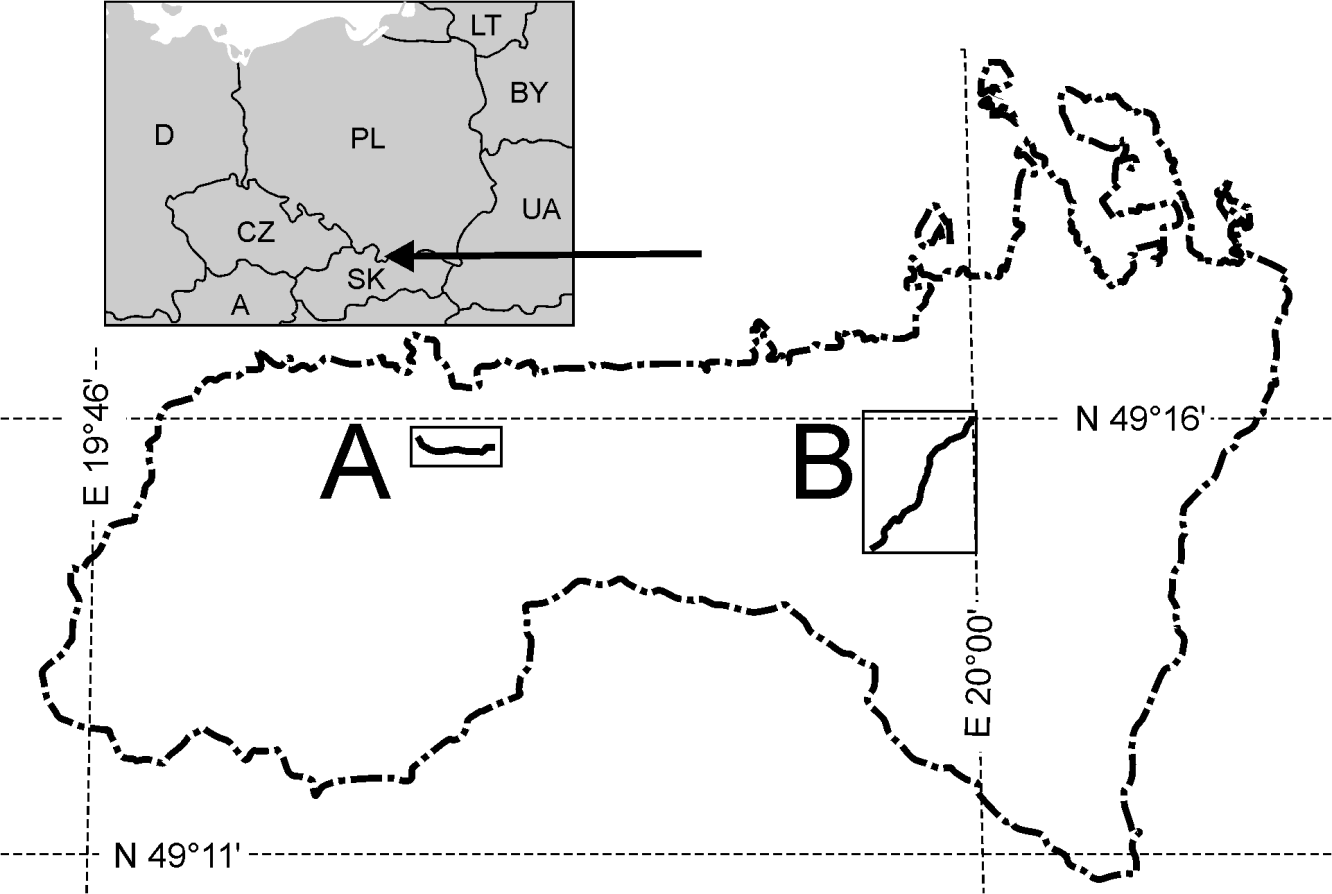
Study plots location in the Tatra National Park. A—"Cyrla-Kopieniec", B—"Hala Gąsienicowa—Rówień Waksmundzka".

Two tourist tracks located in spruce forests of the Polish Tatra National Park were selected for the study, following the consent of the Park authority (Fig 1). The first one, Cyrla-Kopieniec, intersects the lower montane spruce stand in elevation between 1000 to 1200m above sea level. A considerable part of the studied track runs along a ravine where rainwater flows. This was probably the course originally marked out for the track. Currently, two banks are in use where broad paths have been trodden, reaching up to 2m in width in some places. Most of the track is devastated by hiking traffic–soil is largely exposed and tree roots are bared. The ground around the trees is usually circularly trampled. Another factor with a harmful effect on the condition of the track is its direction perpendicular to the slope contours, which facilitates erosion. The second track, Hala Gąsienicowa–Rówień Waksmundzka, traverses upper montane forests, sometimes running along the timber line. It features considerable elevation differences from 1440 m and 1560m above sea level. Approximately 65% of the track length runs along the slope contours and 35% of the route runs perpendicularly, which is clearly visible from the level of soil damage. Most of the track has the form of an unadapted path–narrow, largely muddy, with many bare roots of trees trampled around from one or both sides.

Dendrochronological methods based on the analysis of annual increments were employed in the studies. On each track, four sampling plots were selected. Two zones: damaged and unaffected by tourism, were distinguished in each sampling plot on the based upon visible damage to soil cover and the degree of coverage by vegetation.

Increment cores from 10 spruces (*Picea abies*) were extracted in each sampling plot, which included 5 trees growing in the damaged zone, and 5 trees growing in the zone unaffected by tourism. Assuming that there might be increment reduction attributable to trampling in heavily trampled zones, the following further assumptions were adopted while selecting measurement sites:

— the sample for dendrochronological analysis is homogeneous, i.e. all bored trees belong to one generation, one biosocial class, and grow in the same microhabitat conditions.
— trees growing in all zones are exposed to the same climatic conditions.

According to the permission of the Tatra National Park authority only one increment core was extracted from each tree at the height of approx. 1.30m. The cores were then dried and polished. Increment cores from diseased trees, trees infested with fungi and trees bearing visible anomalies in annual increments were recorded but not included in the analyses. The next step was to scan each sample and mark the annual increments of each tree using CooRecorder software. Subsequently, the distances between increments were determined and saved as a spreadsheet using CDendro software. The next stage of work involved the dating of increment sequences to calendar years derived from the visual synchronization of increment curves and signature years being taken into account. In order to perform a more objective comparison of the data, the obtained results were subjected to normalization with the MS Excel spreadsheet. We calculate the normalized value of all measures based on the parameters (mean, SD) of the all measurements. All further analyses were conducted using normalized variables. Mean annual increments were subsequently calculated for trees growing within and beyond tracks in each year (S1 File). Increment sequences obtained from trees growing in different damage-exposure zones were compared. For calculation the descriptive statistic of the tree ring width, as well as for the building the chronology we have used dplR 1.6.3. package for R 3.0.3. [36,37] (S2 File). The normalized increment trend of trees growing outside the damage zone, i.e. beyond the reach of tourists, was treated as a reference trend. Increment trends of trees from the successive damage zones in each tourist trail were then compared to the reference trend. Such comparisons were made for individual years.

### Assessment of the sample representativeness

The question of how the reduction of sample size may influence the results obtained was analyzed by *iterative simulation*, with its conditions established using the field study results. The simulation involved drawing without replacement from a pool of virtual objects, equal to the number of bored trees. At the first stage, the fraction of drawn trees that had not met the requirements for their inclusion in the analyses–trees with increment anomalies, trees dry-rotten inside etc.–was determined, and then a “flawed” attribute was assigned to the number of objects equal to this fraction. Samples were randomly collected, by drawing without replacement from the population of virtual objects thus prepared, whilst the fraction of objects with the excluding attribute was noted for each sample. The sizes of samples drawn ranged from 5 to 90 objects, increasing by 5 object intervals. Drawing was performed in 5000 replications for each sample size and the fraction of “flawed” trees was counted each time. The simulation formula was prepared using the R statistical package with generator implemented in PPS functions [38,39,40].

### The assessment of tourist impact on increment change dynamics in trees

The effect of the selection of the method to divide the function’s domain was analyzed comparing the linear regression fit illustrating increment changes in trees growing adjacent to tracks and those located beyond the direct impact of tourism, which was performed in two variants: the joint and separate examination of the two study plots. To supplement this analysis, a statistical model from the GLM (Generalized Linear Model) family was added, which includes not only the year, but also the study plot effects and whether the tree grew within the coverage of track impact or belonged to the control group. The effect of the division of function’s domain on the results was tested by the analyses, performed for the Kopieniec study plot, covering the periods before and after an abrupt increase in tourist traffic. Published sources [41] indicate that the rapid intensification of tourist traffic was observed in the Tatra National Park between 1950s and 1980s. However, due to the various popularity of tracks and the tourist profile, it is impossible to strictly and objectively identify the borderline between the periods of low and high traffic intensity. In order to determine how the arbitrary selection of this borderline would influence the results of analyses, they were conducted adopting three different variants of timeline division into periods before and after the start of mass tourist traffic in the Tatra Mountains. The years 1960, 1970 and 1980 were selected as watershed dates. More objective determination of the difference in the tourist regime may be done with the technique proposed by Rodionov (2006). However data about the number of visitors were collected with different time interval [41] in result they can't be treated as the typical time series.

## Results

### Assessment of representativeness

Out of 90 bored trees, 15 –which represents 16,7%–did not meet the requirements for their inclusion in further analyses. The simulation of drawing differently sized samples without replacement was performed on a group of 90 objects, 15 of which had the “flawed” attribute. With small sample sizes and 5000 replications of drawing, the fraction of objects marked as “flawed” significantly deviated from the 16,7% occurring in the population.

**Fig 2:**
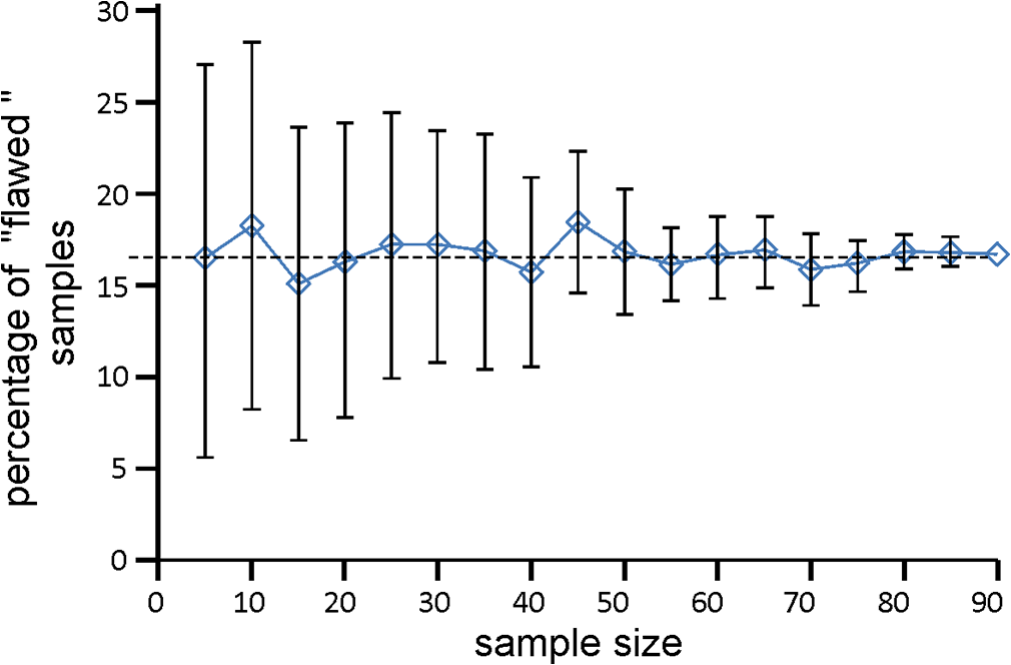
Average percentages of objects with "flawed" attribute received in 5000 draws for different sample sizes.

As the number of objects drawn in a sample grew, the mean fraction calculated for 5000 drawings became increasingly closer to the actual value and the standard error of the estimation became distinctly lower (Fig 2).

### Analysis of the process

**Fig 3:**
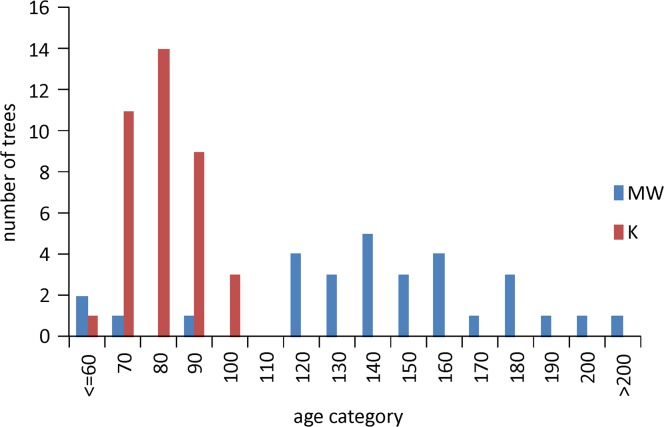
Numbers of trees in subsequent age category analyzed on both tourist tracks.

The analysis included data from 75 spruces (CM Route 37 and 38 of Kopien) iec aged from 59 to 239 years (Fig 3). The descriptive statistic of the sample, as well as the chronology parameters are presented in Table 1.

**Table 1:** Basic statistic, GLK (Gleichläufigkeit) and EPS (expressed population signal) and mean sensitivity of tree time-series of both study plots.

	Murowaniec-Waksmundzka		Kopieniec	
	Track	Control	Track	Control
Studied period	1770–2007		1910–2007	
	1770–2007	1830–2007	1910–2007	1913–2007
Number of bored trees	50		40	
	25	25	20	20
Number of flawed	13		2	
	6	7	1	1
Min TRW mm	0.102		0.132	
	0.102	0.173	0.141	0.132
Max TRW mm	6.515		16.631	
	4.589	6.515	16.631	7.288
Mean TRWmm	0.9264		1.9105	
	0.8521	1.0172	1.8805	1.9422
TRW SD	0.54125		1.20224	
	0.50814	0.56622	1.30386	1.08349
Mean sensivity	0.21		0.26	
	0.19	0.22	0.31	0.20
GLK	0.68		0.56	
	0.72	0.64	0.52	0.58
EPS	0.86		0.93	
	0.91	0.84	0.89	0.95

The analysis of increment changes over years was carried out assuming (testing) two possible process course models: linear and negative exponential, the latter being typical of long-term increment changes [42,43]. The fit of a cloud of points to other curves (logarithmic, polynomial, Weibull) was also checked, with glm 2 1.1.2 and gnm 1.0–8 R package [44]. and the curve characterized by the maximum R^2^ value was subsequently selected (Table 2). In order to determine whether curve selection significantly affects the level of fit to a cloud of points, residuals computed for regression line, negative exponential model and the curve showing the best fit (Table 3) were compared. The comparison of data collected from both study plots indicates that normalized increments of trees growing near the track decrease significantly over time. Fit parameters of the line to the cloud of points were extremely low, both for the linear (R^2^ = 0.043244; f = 17.4467; p<0.0001) and negative exponential (R^2^ = 0.065612; f = 27.1044; p<0.0001) model. Due to such a low level of predictiveness, deriving conclusions from the course of these lines can be considered rather unreliable.

**Table 2:** Fitting parameters for the regression lines calculated for given time periods together with the significance levels for the comparison of the regression and b coefficients.

	function	R^2^	F	p
Kopieniec Tourist track	linear	0.739856	221.8340	<0.0001
	Negative exponential	0.792372	297.6727	<0.0001
	Best fitted	0.862832	321.235	<0.0001
Kopieniec Tourist track Tourist track	linear	0.933769	1099.699	<0.0001
	Negative exponential	0.929365	1026.263	<0.0001
	Best fitted	0.956741	560.2814	<0.0001
Murowaniec-Waksmundzka	linear	0.331232	55.4941	<0.0001
	Negative exponential	0.327015	54.4227	<0.0001
	Best fitted	0.514038	38.7850	<0.0001
Murowaniec-Waksmundzka Reference zone	linear	0.691849	251.4581	<0.0001
	Negative exponential	0.670051	227.441	<0.0001
	Best fitted	0.796942	143.9059	<0.0001

**Table 3:** Comparison of goodness of fit the different functions describing the changes of the increment sizes for each study sites and zones separately.

	Linear vs. negative exponential	Linear vs. best fitted	Negative exponential vs. best fitted
Kopieniec Tourist track	0.2438	0.0281	0.0481
Kopieniec Tourist track Tourist track	0.4124	0.5037	0.4772
Murowaniec-Waksmundzka Tourist track	0.6284	0.0172	0.0147
Murowaniec-Waksmundzka Reference zone	0.6801	0.0364	0.0329

**Fig 4:**
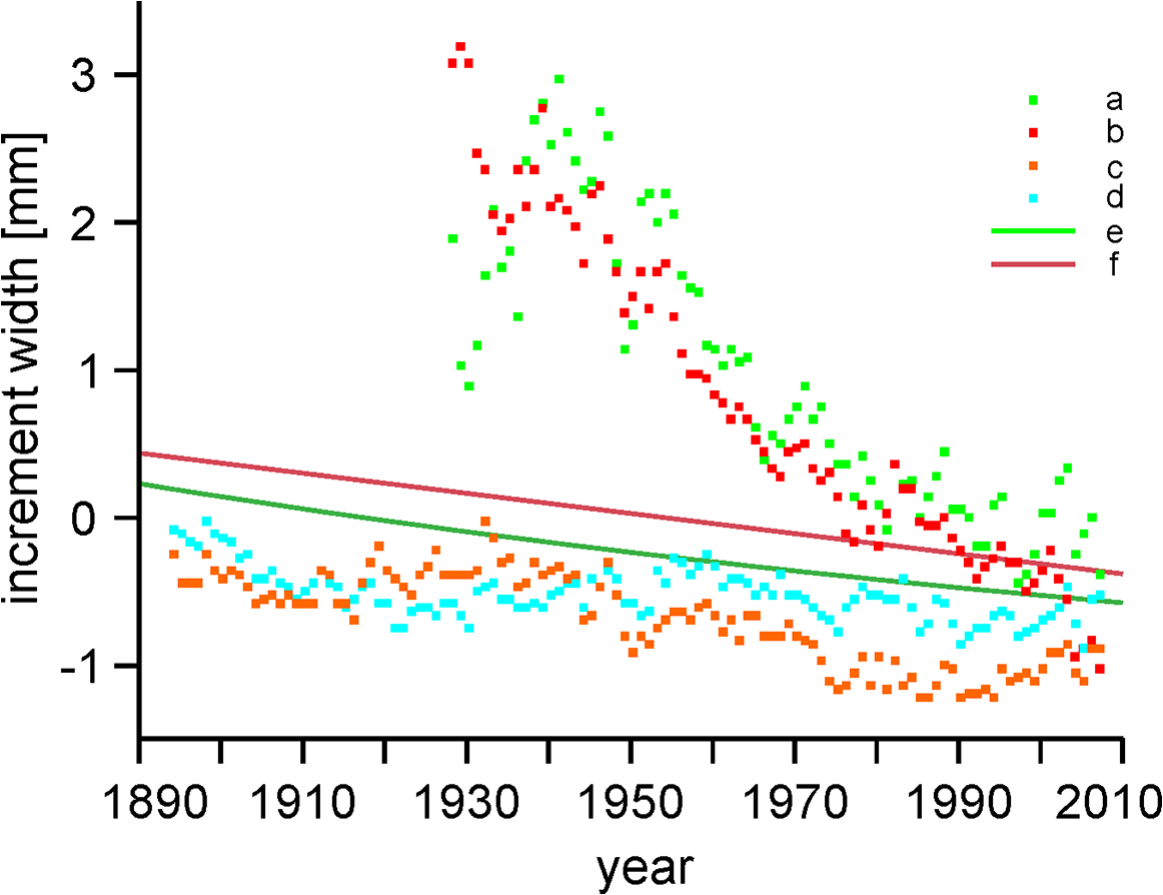
Changes in the increments of trees from the different areas growing near the track and beyond the range of its direct impact. a–average normalized increments of trees from the control zone in the "Cyrla-Kopieniec" study plot, b–average normalized increments of trees growing near the track in the "Cyrla-Kopieniec" study plot, c–average normalized increments of trees growing near the track in the "Hala Gąsienicowa–Rówień Waksmundzka" study plot, d–average normalized increments of trees in the control zone in the "Hala Gąsienicowa–Rówień Waksmundzka" study plot. e–regression line for tree increment changes, f–negative exponential fit for tree increment.

**Fig 5:**
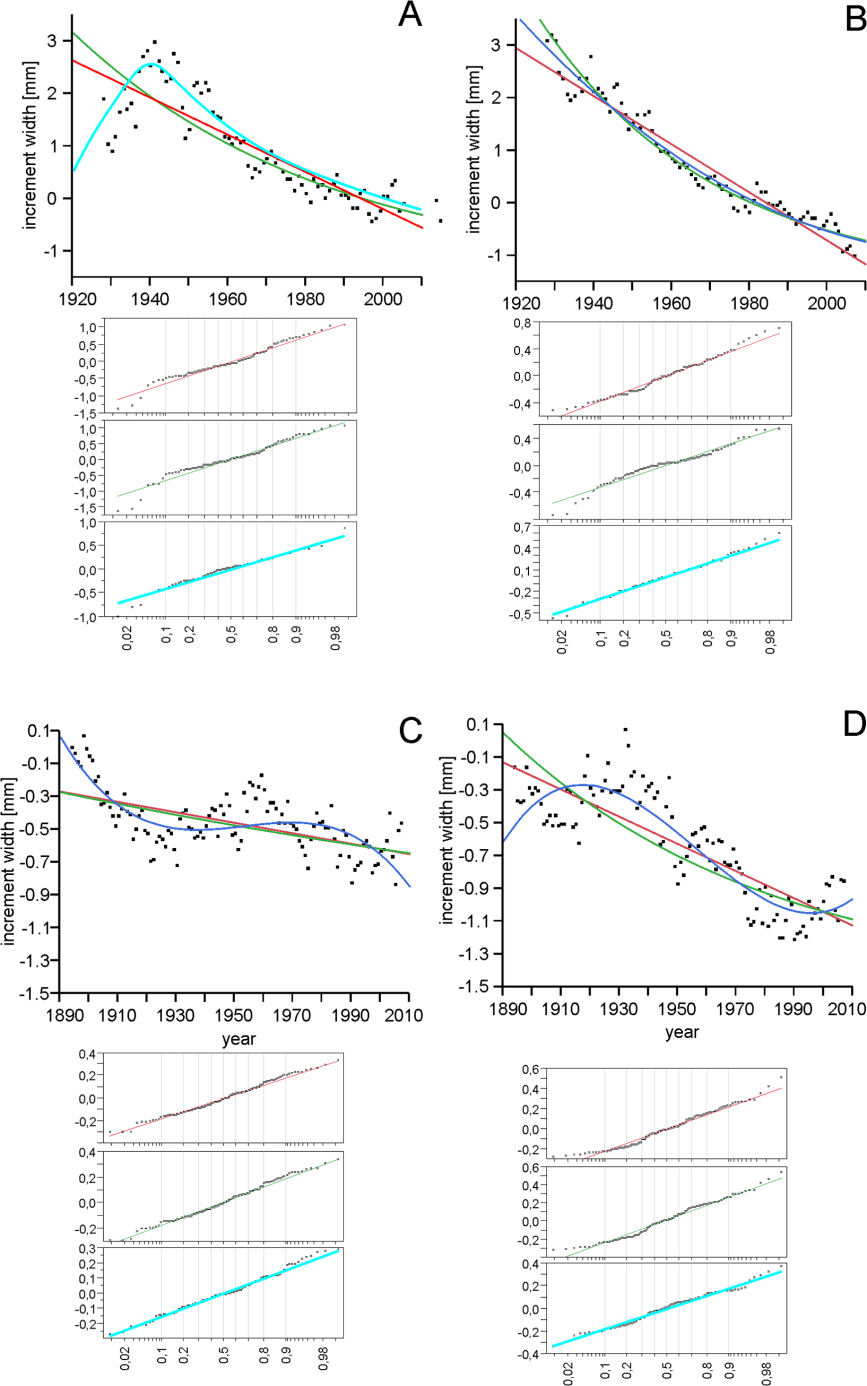
Comparison of lines showing the course of processes for different study plots. A–trees growing in the control zone in the "Cyrla-Kopieniec" study plot, B–trees growing near the track in the "Cyrla-Kopieniec" study plot, C–trees growing in the control zone beyond the track in the "Hala Gąsienicowa–Rówień Waksmundzka" study plot, D–trees growing near the track in the "Hala Gąsienicowa–Rówień Waksmundzka" study plot. 1 –linear fit, 2 –negative exponential fit, 3 –best-fitted curve: for A–negative exponential, B–Weibull fit, C and D– 3rd degree polynominal. Below the main charts q-q plots for each fitted curves are presented.

Here it should be stressed that a strong difference between the studied areas became evident (Fig 4), which is demonstrated by the distinct differences in the values of residuals for both areas, calculated both for the linear (t = 22.0664; p<0.0001) and negative exponential (t = 24.2186; p<0.0001) model. Therefore, we have decided to consider the course of processes separately for each study plot (Table 2, Fig 5). Not only the major difference between the study plots is conspicuous, but also the fact that the process of change occurring in each of them is best described by different models. In the case of the Kopieniec track, linear fit does not significantly deviate from the negative exponential and Weibull fits (Table 3), whereas for the control area, the level of Weibull fit was significantly higher than the linear and negative exponential. On the other hand, as regards the Murowaniec-Waksmundzka section, the best fit–significantly higher than the linear and negative exponential–both for trees growing near the track and in the reference area, was shown by the line representing a 3rd degree polynomial function (Tables 2 and 3). In this case, the parallelism of these lines was compared [45], demonstrating significant differences (f = 2.0896; p = 0.0053).

A multifactor analysis, covering the impact of the study plot and the position of a tree, in the direct vicinity of the track or within the control group, on the increment rate, has demonstrated that the GLM model, assuming a normal distribution of the dependent variable and featuring an identity link function, shows significant fit (χ^2^ = 478.628; df = 3; p<0.0001). Analysis of the impact of individual parameters shows in turn that both the sampling plot and the position in relation to the track play an important role.

The analysis of changes in increment size in different time periods indicates that the applied method of timeline division into periods before and after the start of mass tourist traffic had a significant influence on both slope coefficients and linear regression fit parameters (Table 4, Fig 4).

**Table 4:** Parameter estimation for the statistical model of the common influence of the year, study plot and the direct impact of the tourist track on the tree ring size. The factor “zone” represents the part of the study plot under tramping influence or the control one.

Term	Estimate	Estimation SE	χ^2^	p
Intercept	29.898115	1.8359707	202.09436	<0.0001
Study plot [Kopieniec]	0.855213	0.0289686	456.87314	<0.0001
Zone [control]	0.084999	0.0274209	9.4916453	0.0021
year	-0.014431	0.0009371	185.06683	<0.0001

## Discussion

The presented results clearly show that methodological details may significantly influence the results obtained, which occurs already at the stage of sampling. Due to the limitation of the number of increment cores imposed by nature protection constraints, it might be impossible to obtain a sample that meets the *least significant number* requirements in the case of high variation of obtained results. In these circumstances, paradoxically, sample representativeness might be reached by the researcher taking decisions with some degree of arbitrariness when selecting trees for the study instead of sticking to random selection methods. This reduce the probability to take a sample from an atypical specimen, such as a young or damaged tree, being drawn and the parameters of a small sample being thereby significantly affected. In our case, this problem regards trees whose internal structure made the samples useless for analysis (Table 1). The position of the trees close to a tourist route, compared to trees positioned beyond its influence (Chi square = 1.23*10^31^; p = 1) with the significant differences of the study plots (Chi square = 7.056; p = 0.02930) points to the conclusion that a factor relating to tourism is not responsible.

If there are different probability of influence the analyzed factor, the stratified samples methods may be helpful. In our research the zone of trampling impact play the role of the “strata”–in whole study plots trees under direct trampling pressure are of course less numerous.

The hypothesis that results can be distorted by the differences between study plots was also supported by obtained results. Multifactor analysis techniques can be applied to determine whether samples obtained in different plots should be examined jointly or separately–a significant effect of the sampling plot suggests that they should be studied separately. From the viewpoint of pure statistical methods [46], the use of multifactor analyses is considered a more advisable approach, however–particularly when categorical predictor variables are taken into account–the obtained statistical models are marked by low predictability, although they do explain the mechanisms of processes.

**Fig 6:**
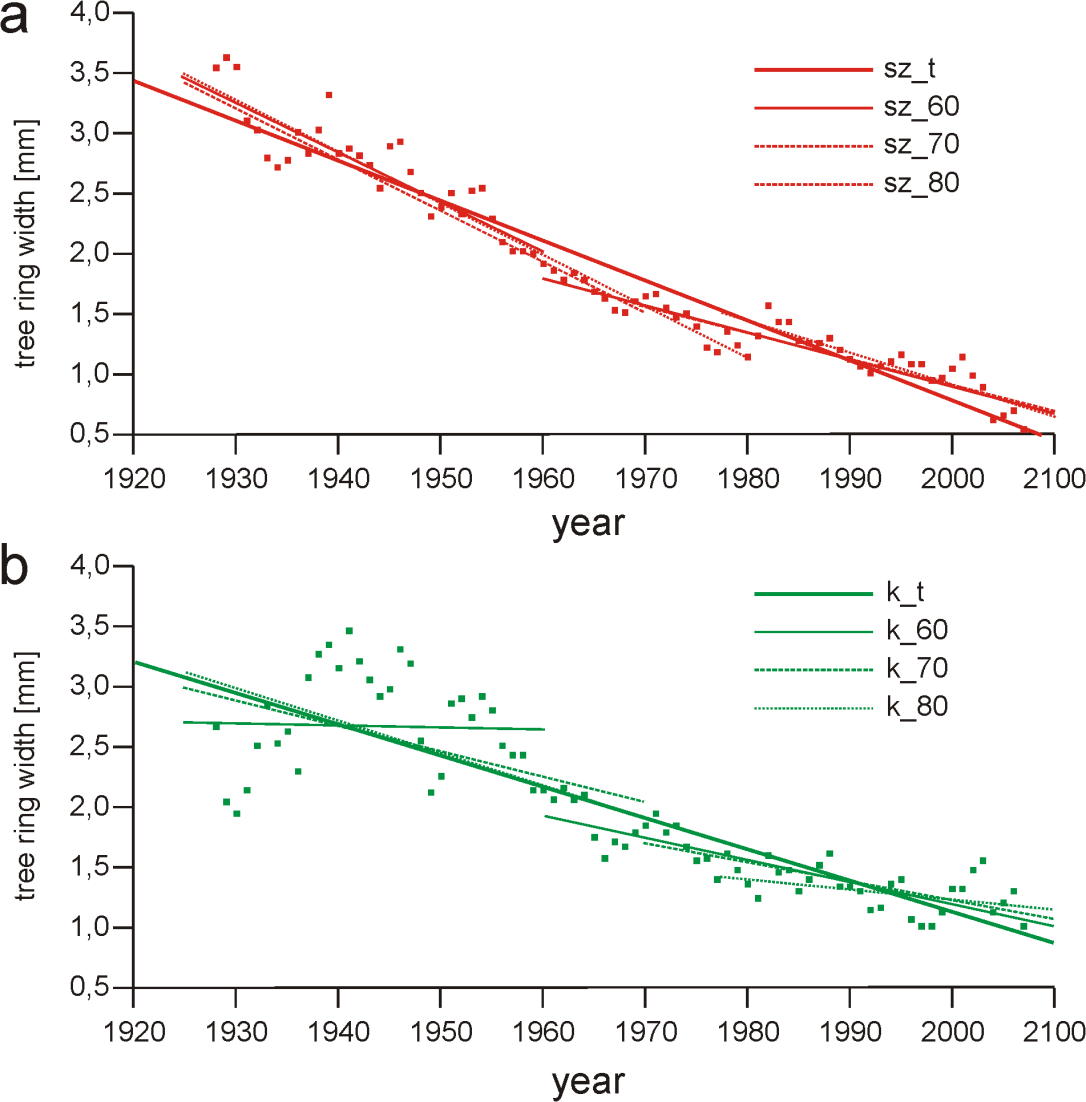
Regression lines of the relationship between year and the size of the tree ring. (a) trees growing in the vicinity of the tourist track and (b) trees growing beyond the scope of the track impact calculated for the whole growing period (sz_t, k_t) as well as separately for the periods before and after the breaking points located in 1960 (sz_60, k_60), 1970 (sz_70, k_70) and 1980, (sz_80, k_80).

Because increment change tendencies are usually curvilinear, comparing linear regression coefficients may result in the process being distorted. One solution to this problem is to divide the analyzed time period into sections and analyze them individually (divide the domain of the time function of increment size) [35](Fig 6).

However, the above-mentioned method is subject to major restraint, i.e. the necessity to establish borders between the analyzed periods, as shown by this study. Given the lack of specified methods, these periods are marked out in a way that is largely arbitrary, whereas even slight modifications may produce dissimilar results, as shown by the analyses. The application of curvilinear regressions seems to be a solution in this situation. However, this approach is not free of difficulties either. If the course of the process is described by a polynomial equation, one may expect a shift of the characteristic elements of the function (extrema, inflection points) in relation to the real events on the time axis. The probability of such a shift is highest when the function has low goodness of fit or when single points with large *residuals* are present. The necessity to conduct an analysis of the function is another technical hindrance. This problem can be solved by applying *cubic spline* fits, which divide the function’s domain in an objective way. Regrettably, apart from the issue of arbitrary selection of the function’s level of fit, domain splitting makes the function considerably harder to analyze, especially that the commonly used statistical packages do not provide full information concerning the formulas of functions fitted using the q-s method [27]. The solution in this situation may entail fitting the course to several functions most typical of a given process. As a function choice criterion, we suggest taking a conservative approach, i.e. treating a linear function as a starting-point function, and conducting the analysis of other models of the course only in cases when *residuals’ absolute values* calculated for the course are significantly higher than for the linear function. If processes are described by the same curvilinear functions, they may be compared using parallelism analyses developed by Huet [45].

## Conclusions

Dendrochronological methods represent a promising tool in the research of the long-term impact of tourist traffic on tree stands, although their application is subject to a number of snags that can significantly affect results.

Nature conservation regulations compel the research to be conducted using small samples, which requires paying particular attention to ensure their representativeness. In certain situations, this may entail abandoning random selection methods, considered the most objective, and resorting to arbitrary decisions taken by the researcher when selecting specimens for core extraction.

The processes of increment rate changes over time may be determined not only by the level of tourist pressure but also by other environmental factors. If these interdependencies are very clear, it is advisable to separately analyze the phenomena occurring in different fragments of the study area.

When a process has a non-linear character, it seems advisable to apply analyses other than linear regression. Although this results in a more complex data analysis process, the alternative method involving the division of time axis into sections is highly prone to artifacts.

## Supporting Information

S1 File Data collected during the field research. The file consist two tables. Table **"RD"** includes data about tree ring width [mm]. The rows represents particular years, variables trees described according following scheme: "StudyPlot_Zone_TreeNr" where: K-"Kopieniec"," MW-"Murowaniec-Waksmundzka"; "T"- trampling zone; "C"- control zone. Empty variables represent flawed trees. Table **"Norm"** includes normalized average annual increments for trees growing under trampling impact and at the reference area, for both analyzed study plots.
(XLS)

S2 File R codes. R codes used by authors during the data analysis.
(R) Click here for additional data file.

## References

[1] Liddle MJ. Recreation ecology: effects of trampling on plants and corals. Trends in ecology & evolution. 1991; 6 (1): 13–17. 21232413 10.1016/0169-5347(91)90141-J

[2] Liddle MJ. Recreation ecology: the ecological impact of outdoor recreation and ecotourism Chapman & Hall Ltd, Melbourne London 1997.

[3] Freitag R, Pyka D. Global Tourism in 2007 and beyond—World Travel Monitor's Basic Figures In: Conrady R, Buck M. editors. Trends and Issues in Global Tourism 2008. Springer—Verlag, Heidelberg, Berlin; 2008 pp. 3–26

[4] Reiter A. Eco-leadership and Green Lifestyle: Successful Strategy for a Growing Market Segment? Trends and Issues in Global Tourism. 2011: 93–98

[5] Zaremba D. Ekoturystyka. PWN Warszawa 2007.

[6] Font X, Tiribe J. Recreation, Conservation, and Timber Production: A sustainable relationship? In: Font X, Tiribe J. editors. Forest Tourism and Recreation case studies in environmental management. CABI Publishing London; 1999 pp. 87–102

[7] Skov-Petersen H, Goossen M. Assesing and planning the supply of opportunities for forest recreation and nature tourism In: Bell S., Simpson M., Tyrväinen L., Sievänen T., Pröbstl U. editors. European Forest Recreation and Tourism: A Handbook. Taylor & Francis, Abingdon 2009

[8] Makomaska-Juchiewicz M, Tworek S. Ekologiczna sieć Natura 2000-problem czy szansa Instytut Ochrony Przyrody PAN 2003.

[9] Viljoen JH, Naicker K. Nature based tourism and communal land: The Mavahulani experience. Development southern Africa. 2000; 17(1): 135–146

[10] Kim MK, Daigle JJ. Monitoring of Vegetation Impact Due to Trampling on Cadillac Mountain Summit Using High Spatial Resolution Remote Sensing Data Sets. Environmental Management. 2012; 50: 956–968. 10.1007/s00267-012-9905-7 22930327

[11] Łajczak A. Oddziaływanie narciarstwa zjazdowego i turystyki pieszej na erozję gleby w obszarze podszczytowym Pilska. Studia Naturae. 1996; 41:131–159

[12] Quinn NW, Morgan RPC, Smith AJ. Simulation of Soil Erosion Induced by Human Trampling. Journal of Environmental Management. 1980; 10: 155–165

[13] Becken S, Patterson M. Measuring national carbon dioxide emissions from tourism as a key step towards achieving sustainable tourism. Journal of Sustainable Tourism. 2006; 14 (4): 323–338

[14] Buckley RC. Sustainable tourism: Technical issues and information needs. Annals of Tourism Research. 1996; 23: 925–928.

[15] Martin-Cejas R, Sanchez P. Ecological footprint analysis of road transport related to tourism activity: The case for Lanzarote Island. Tourism Management. 2010; 31(1): 98–103.

[16] Fantucci RM, Sorriso-Valvo M. Dendrogeomorphological analysis of a slope near Lago, Calabria (Italy). Geomorphology. 1999; 30: 165–174

[17] Malik I, Wistuba M, Stopka R, Trąbka K. Rzeźbotwórcza rola wezbrań o różnej wielkości zapisana w anatomii drewna drzew, przykład z Hrubégo Jeseníka(Sudety Wschodnie). Studia i Materiały CEPL, Rogów. 2012; 14 (1): 157–165

[18] Fritts H.C. Tree Rings and Climate. London, New York and San Francisco, Academic Press, 1976.

[19] Helama S, Lindholm M, Timonen M, Eronen M. Detection of climate signal in dendrochronological data analysis: a comparison of tree-ring standardization methods. Theoretical and Applied Climatology. 2004; 79, 3–4: 239–254

[20] Schweingruber FH. Tree rings and environment: dendroecology Paul Haupt AG Bern 1996.

[21] Speer J.H. Fundamentals of Tree Ring Research. University of Arizona Press 2010.

[22] Stoffel M, Ličvre I, Monbaron M, Perret F, Perret S. Seasonal timing of rockfall activity on a forested slope at Täschgufer (Swiss Alps)–dendrochronological approach. Z. Geomorph. NF. 2005; 49: 89–106

[23] Stoffel M, Butler DR, Corona C. Mass movements and tree rings: A guide to dendrogeomorphic field sampling and dating. Geomorphology. 2013; 200:106–120

[24] Ciapała S, Adamski P, Zielonka T. 2014. Tree ring analysis as an indicator of environmental changes caused by tourist trampling–a potential method for the assessment of the impact of tourists. Geochronometria. 2014; 41(4): 392–399 10.2478/s13386-013-0170-1

[25] Łomnicki A. Wprowadzenie do statystyki dla przyrodników Wydanie 4 poprawione i rozszerzone. PWN Warszawa 2010

[26] Plucińska A, Pluciński E. Probabilistyka: rachunek prawdopodobieństwa, statystyka matematyczna, procesy stochastyczne WNT Warszawa 2006.

[27] Sall J, Creighton L, Lehmann A. JMP Start Statistics: A Guide to Statistics and Data Analysis Using Jmp, Fourth Edition. SAS Press Series. (Cary, North Carolina). 2005.

[28] Sokal RR, Rohlf FJ. Biometry The The Principles and Practices of Statistics in Biological Research. WH Freeman & Co. New York 1995.

[29] Armitage P, Berry G, Matthews JNS. Statistical Methods in Medical Research, Fourth Edition Blackwell Science, Oxford; 1994

[30] Israel GD. Sampling the Evidence of Extension Program Impact. Program Evaluation and Organizational Development, IFAS, University of Florida. PEOD-5; 1992

[31] Kraemer HC, Thiemann S. How Many Subjects? Statistical Power Analysis in Research. CA Newbury Park. 1987

[32] Sporek K, Sporek M. Doświadczalnictwo ekologiczne—metody wybrane Wydawnictwo Uniwersytetu Opolskiego Opole 2008.

[33] Cortina JM. What is coefficient alpha? An examination of theory and applications. Journal of Applied Psychology. 1993; 78 (1): 98–104

[34] Ciapała S, Zielonka T. Applicability of the Dendrochronology in the Evaluation of the Long-Term Impact of Hiking on the Condition of Stands Adjacent to Hiking Trails. Folia Turistica 2013; 28(2): 227–236

[35] Kaczka RJ, Wiórkowski S, Czajka B, Skrzydłowski T. Dynamika wzrostu i wrażliwość klimatyczna jodły pospolitej (Abies alba Mill.) w naturalnym lesie dolnoreglowym w Tatrach Polskich. Stud. i Mat. CEPL Rogów. 2012; 1(30): 111–117.

[36] Bunn A.G. A dendrochronology program library in R (dplR).” Dendrochronologia. 2008; 26(2): 115–124. ISSN 11257865. doi: 10.1016/j.dendro. 2008.01.002 http://linkinghub.elsevier.com/retrieve/pii/S1125786508000350.

[37] Bunn A, Korpela M, Biondi F, Campelo F, Mérian P, Qeadan F et al. 2015 Package ‘dplR’ http://cran.r-project.org/web/packages/dplR/dplR.pdf

[38] Crowley MJ. The R Book. John Wiley & Sons Ltd. West Sussex; 2007

[39] R Core Team. R Language Definition. 2013.

[40] Gambio J. Functions for PPS sampling. 2005. ftp://ftp.uni-bayreuth.de/pub/math/statlib/R/CRAN/doc/packages/pps.pdf

[41] Czochański JT. Ruch turystyczny w Tatrzańskim Parku Narodowym IN: Partyka J, (ed) Użytkowanie Turystyczne Parków Narodowych. Ruch Turystyczny—Zagospodarowanie—konflikty—zagrożenia. IOP PAN, OPN, Ojców; 2002 pp. 385–404.

[42] Cook E.R., Kairuiuksitis L.A. (eds). Methods of Dendrochronology Applications in the Environmental Sciences. Kluwer Academic Publishers Dordrecht 1990.

[43] Wimmer R. Wood anatomical features in tree-rings as indicators of environmental change. Dendrochronologia. 2002; 20(1–2): 21–36.

[44] Friedman J, Hastie T, Tibshirani R. Regularization Paths for Generalized Linear Models via Coordinate Descent. Journal of Statistical Software. 2010; 33(1): 1–22 http://www.jstatsoft.org/v33/i01/. 20808728 PMC2929880

[45] Huet S. ed. Statistical tools for nonlinear regression: a practical guide with S-PLUS and R examples Springer Science & Business Media, 2004

[46] Manley BFJ. Multivariate Statistical Methods. A primer Second edition Chapman & Hall, London 1994.

